# Supplementary material for: ML-based detection of depressive profile through voice analysis in WhatsApp™ audio messages of Brazilian Portuguese Speakers
Source: PLOS Ment Health. 2026 Jan 21;3(1):e0000357. doi: 10.1371/journal.pmen.0000357 (PMC12822941; doi:10.1371/journal.pmen.0000357)
Supplement: S1 Text — This file presents detailed statistical comparisons of classification models by AUC values, p-values, Cohen’s d, and percentage differences for each speech task (“How their past week was” and “Counting from 1 to 10”), stratified by gender (female and male). The file also includes interpretation legends for significance and effect sizes. (DOCX) [file pmen.0000357.s001.docx]

**Confidence Matrix (Absolute) - “Counting from 1 to 10” - Female**

**
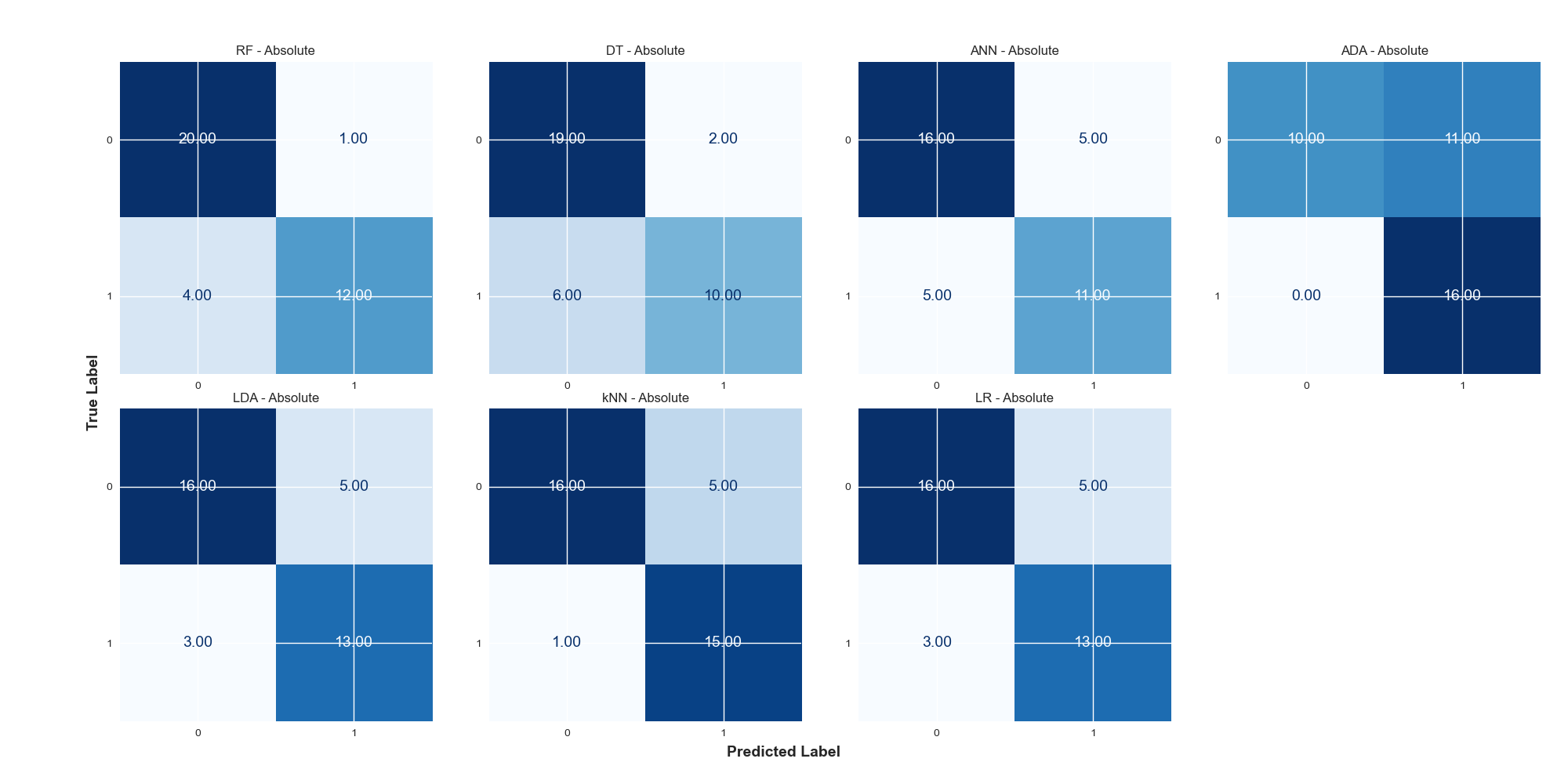
**

**Confidence Matrix (Normalized) - “Counting from 1 to 10” - Female**

**
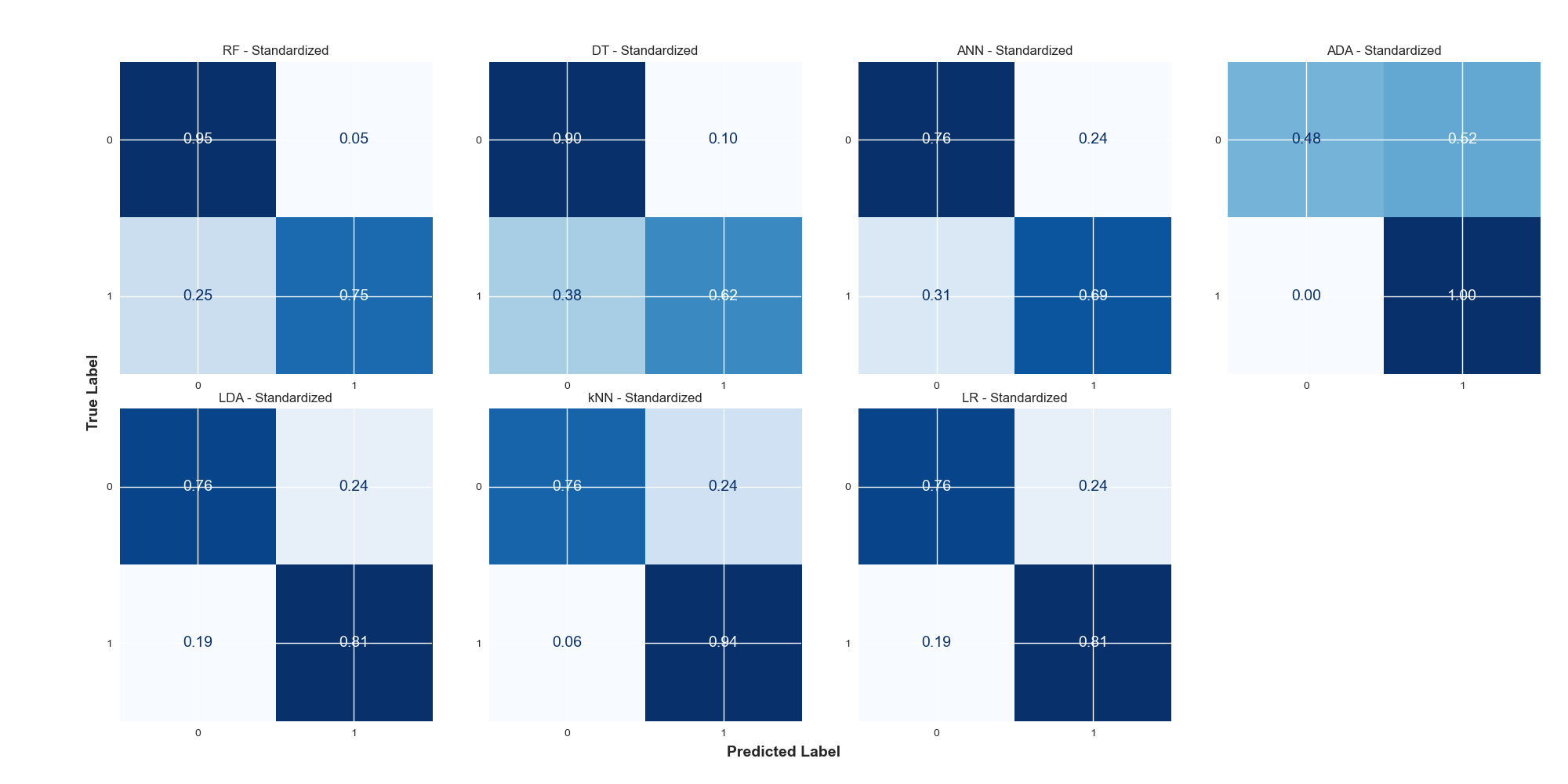
**

**Confidence Matrix (Absolute) - “How their past week was” - Female**

**
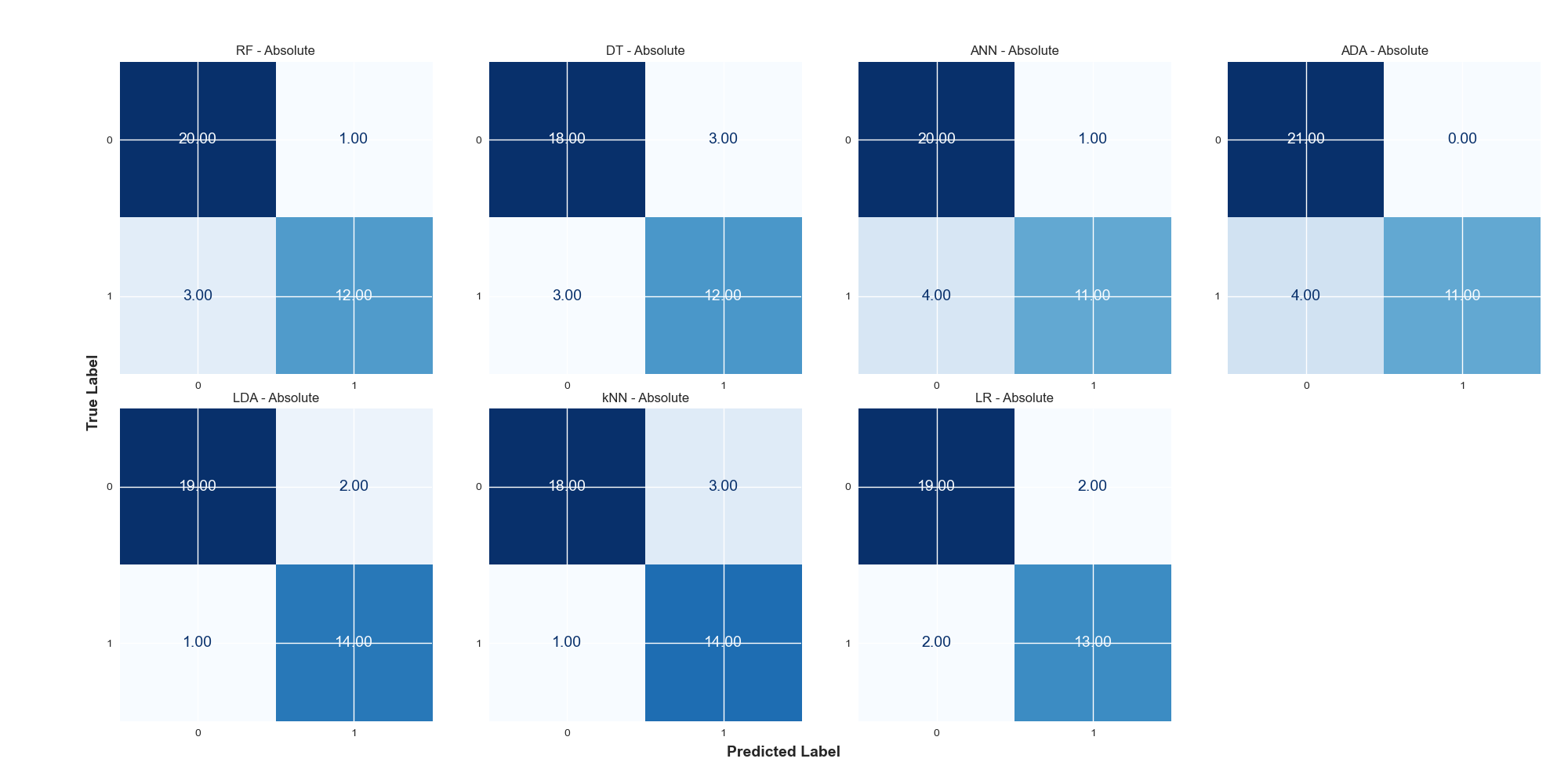
**

**Confidence Matrix (Normalized) - “How their past week was” - Female**

**
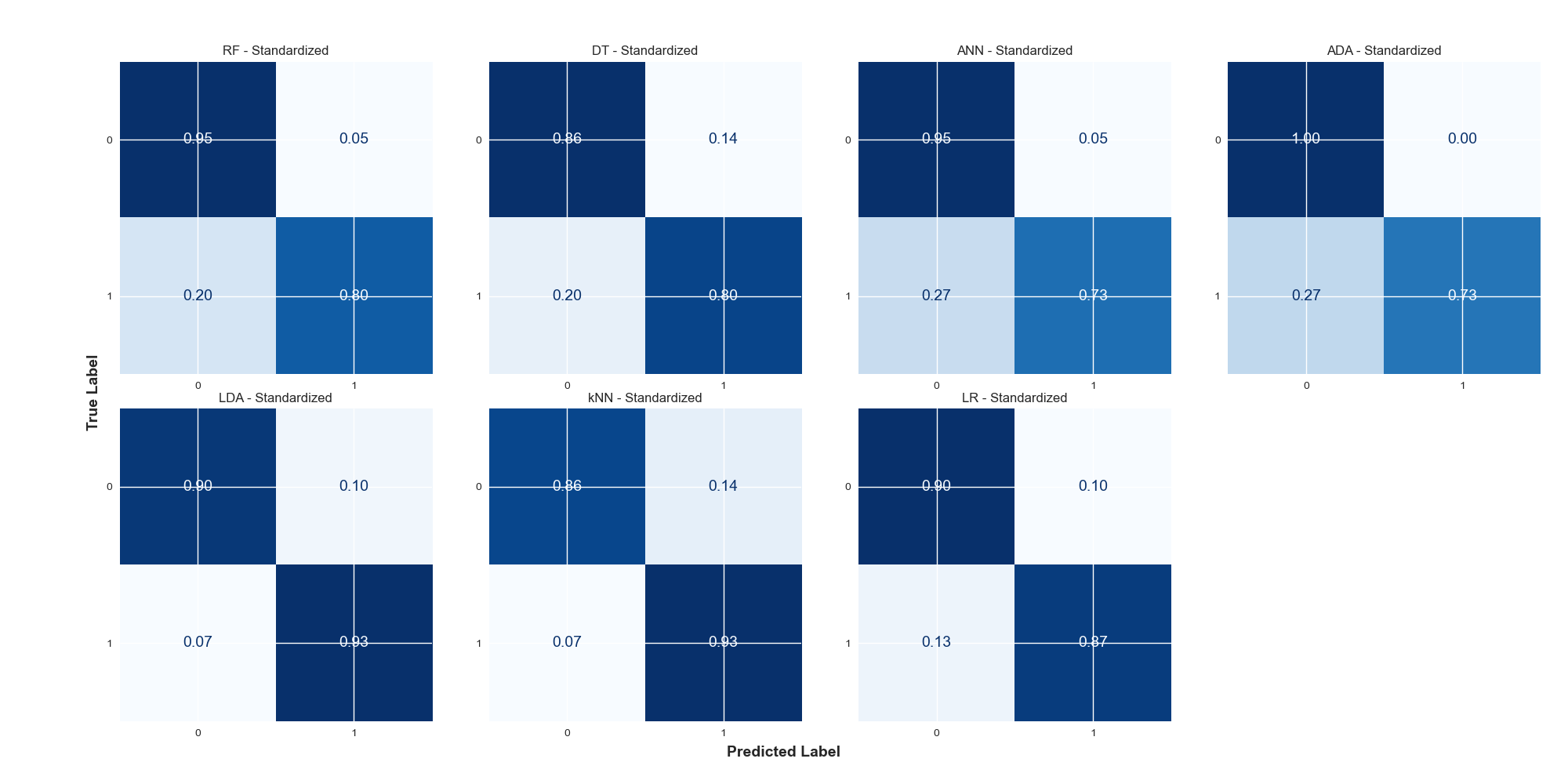
**

**Confidence Matrix (Absolute) - “Counting from 1 to 10” - Male**

**
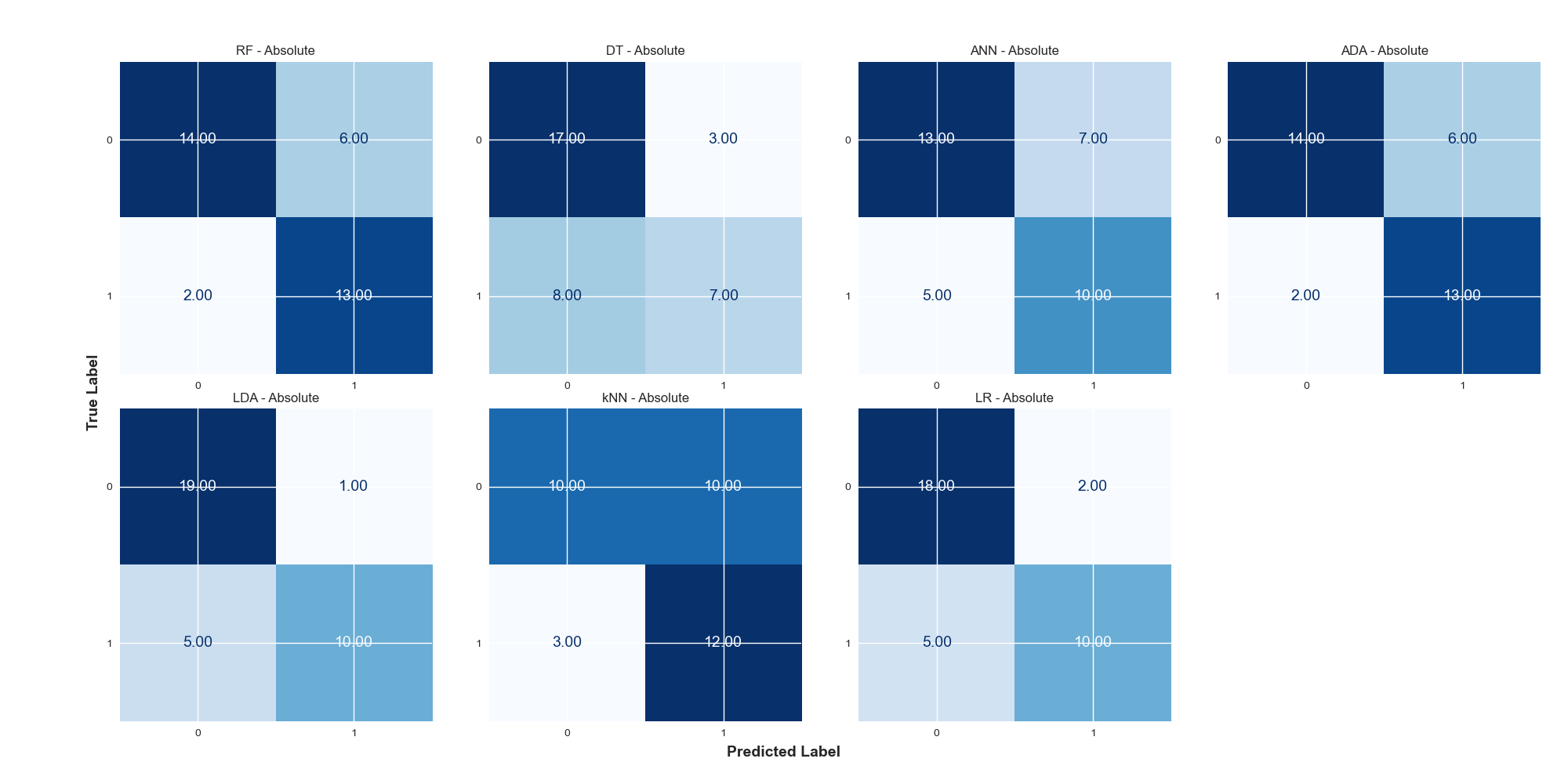
**

**Confidence Matrix (Normalized) - “Counting from 1 to 10” - Male**

**
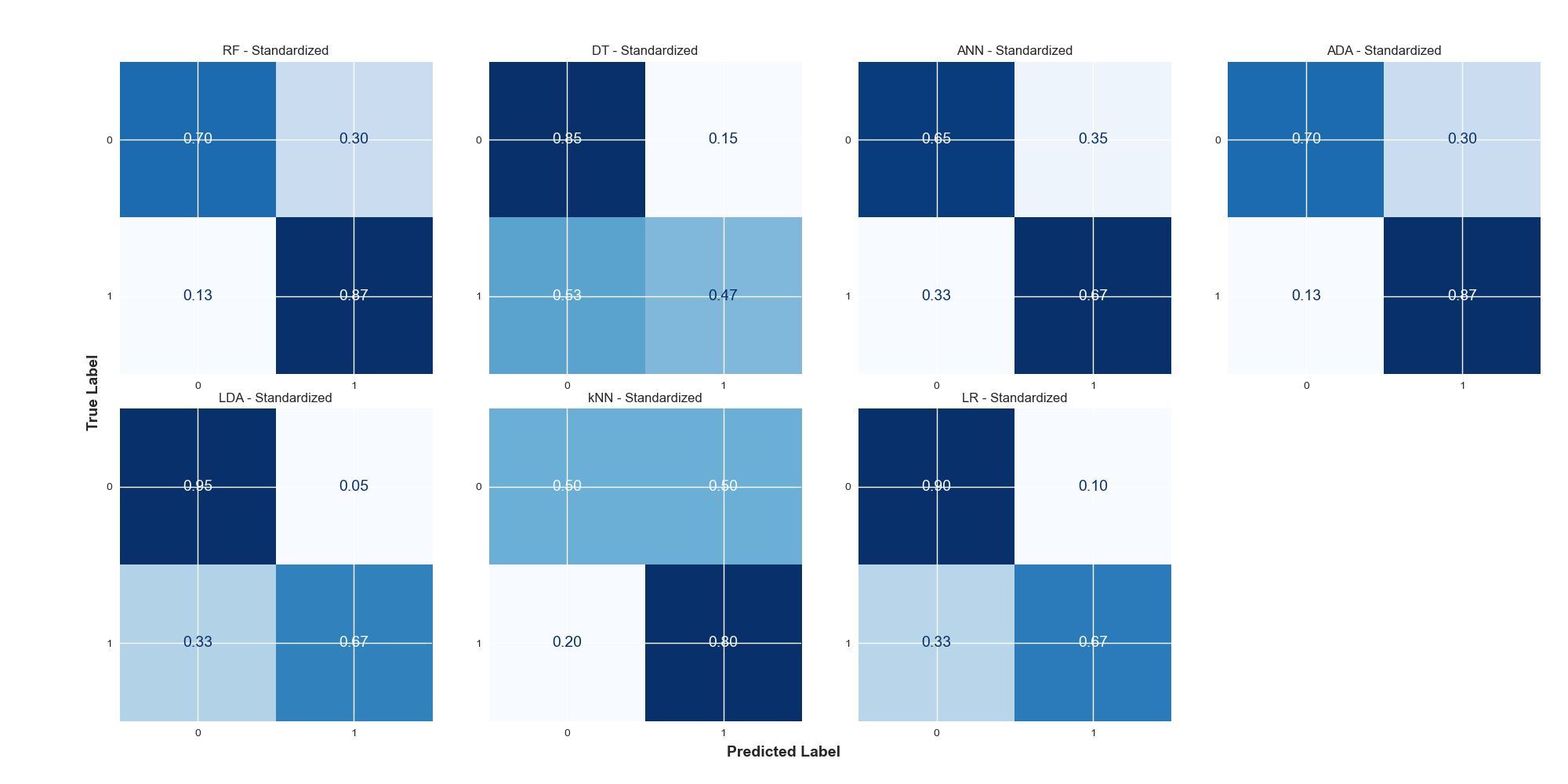
**

**Confidence Matrix (Absolute) - “How their past week was” - Male**

**
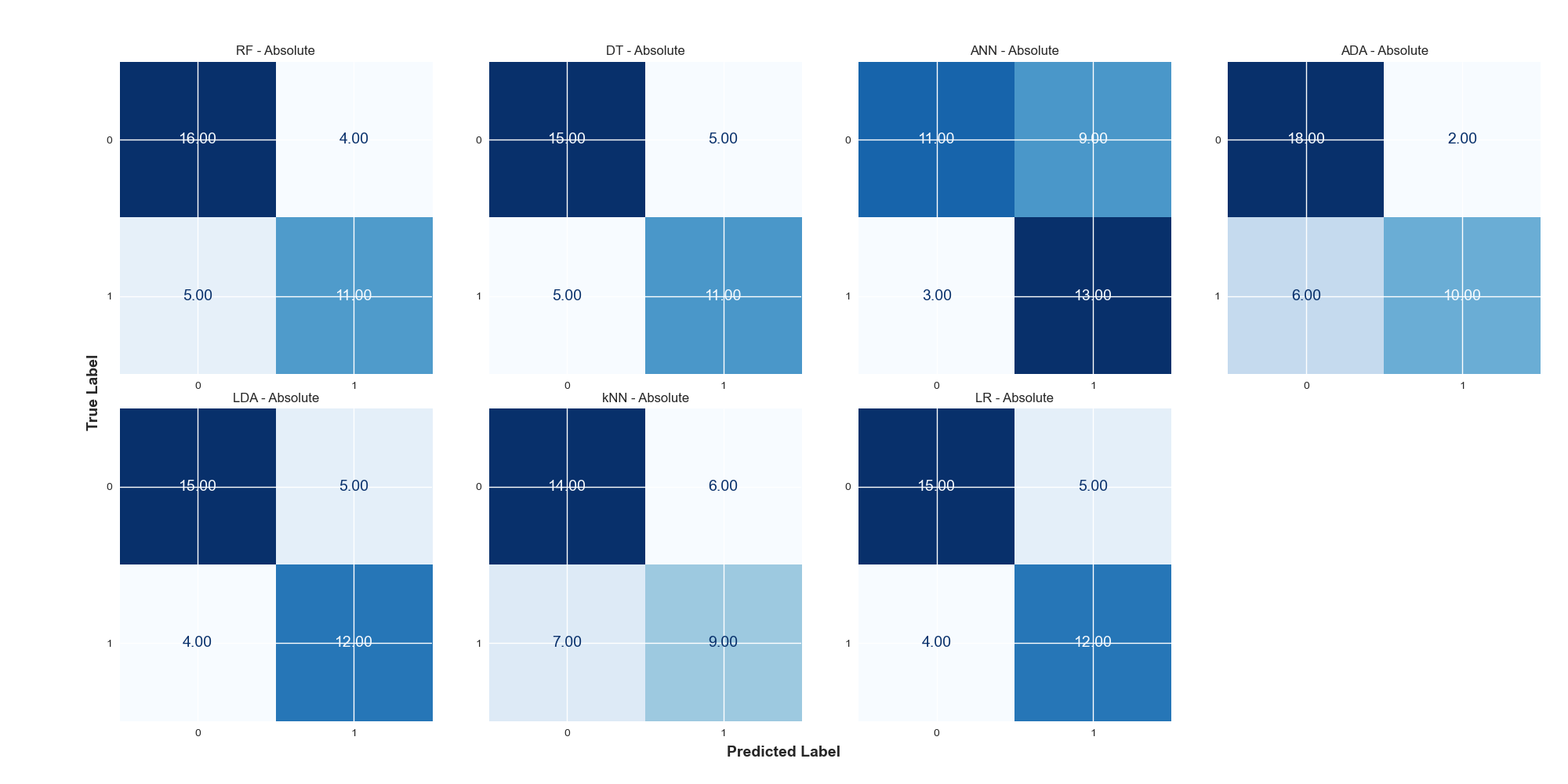
**

**Confidence Matrix (Normalized) - “How their past week was” - Male**

**
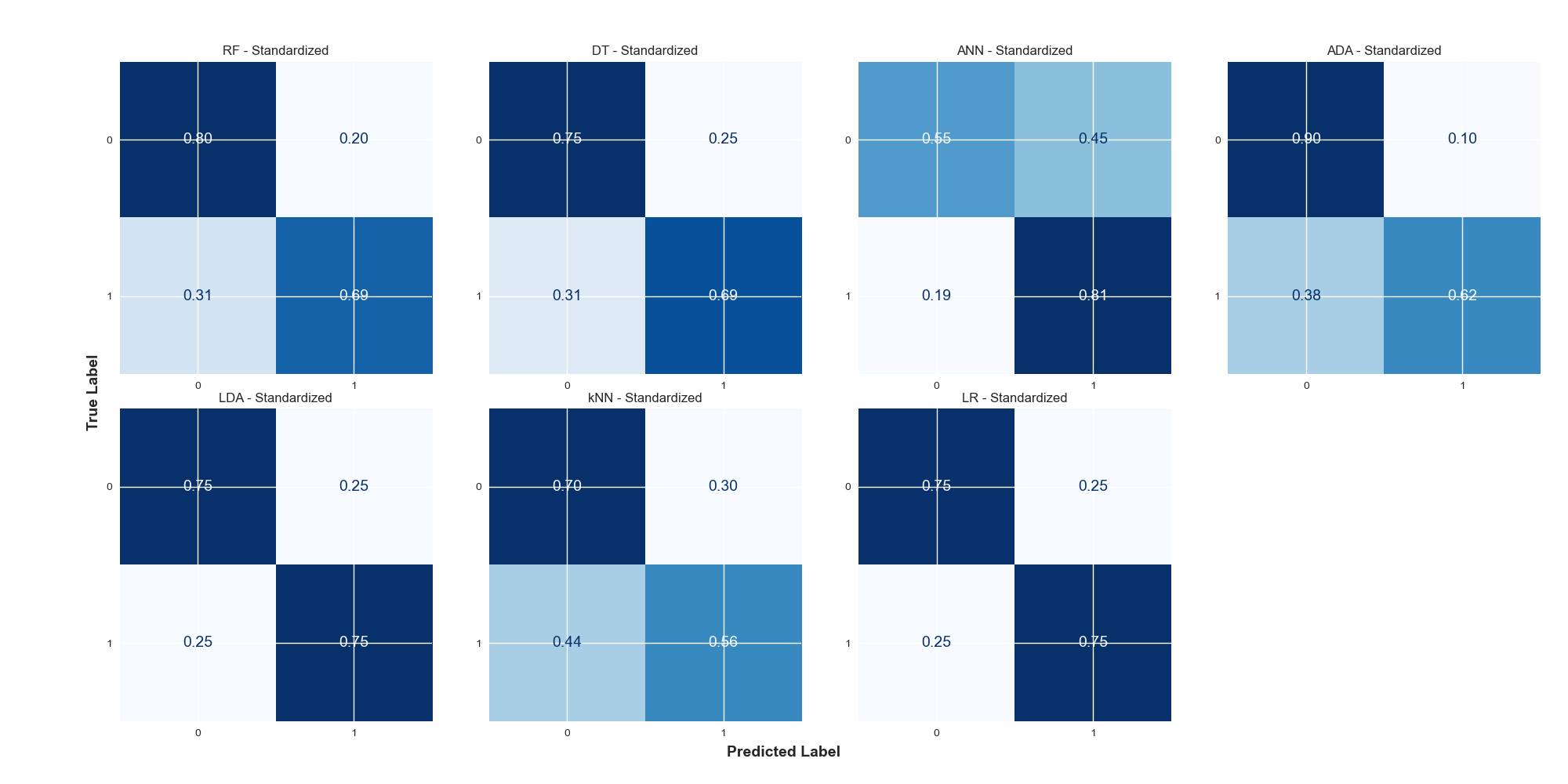
**
