# Supplementary material for: ML-based detection of depressive profile through voice analysis in WhatsApp™ audio messages of Brazilian Portuguese Speakers
Source: PLOS Ment Health. 2026 Jan 21;3(1):e0000357. doi: 10.1371/journal.pmen.0000357 (PMC12822941; doi:10.1371/journal.pmen.0000357)
Supplement: S2 Text — Contains comprehensive performance tables for all tested models (AdaBoost, ANN, Decision Tree, kNN, LDA, Logistic Regression, Random Forest) including recall, specificity, PPV, NPV, accuracy, F1-score, AUC, and error rates with confidence intervals. The results are shown separately for female and male participants across both speech tasks. (DOCX) [file pmen.0000357.s002.docx]

**ROC - “Counting from 1 to 10” - Female**

**
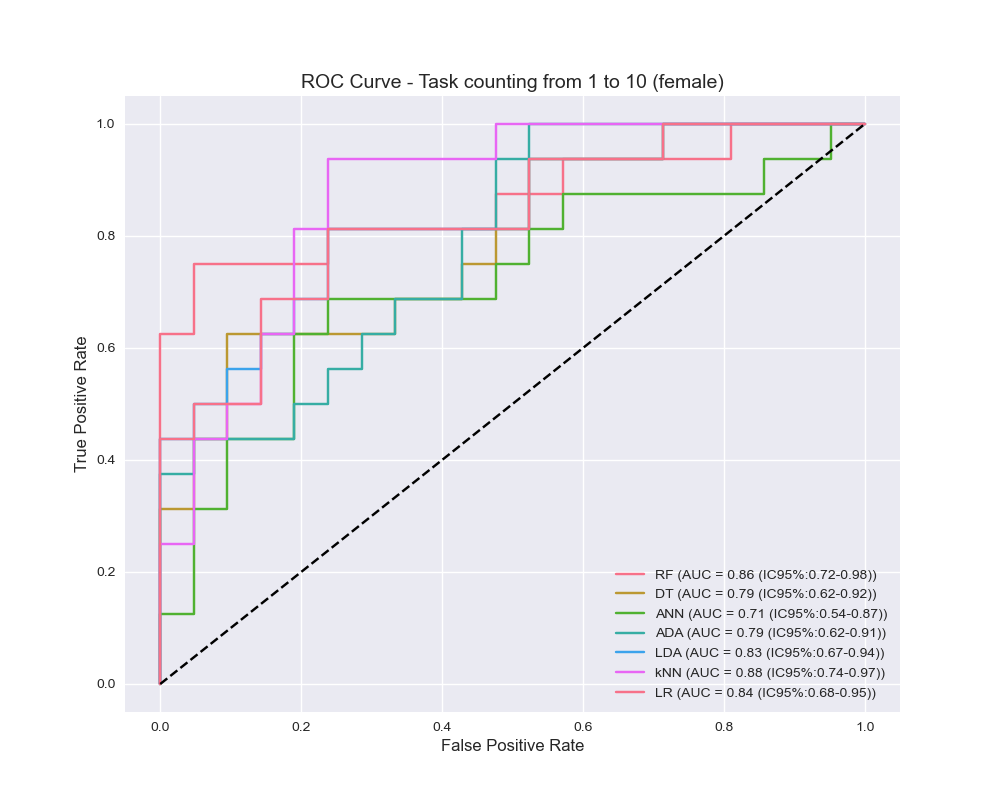
**

**ROC - “How their past week was” - Female
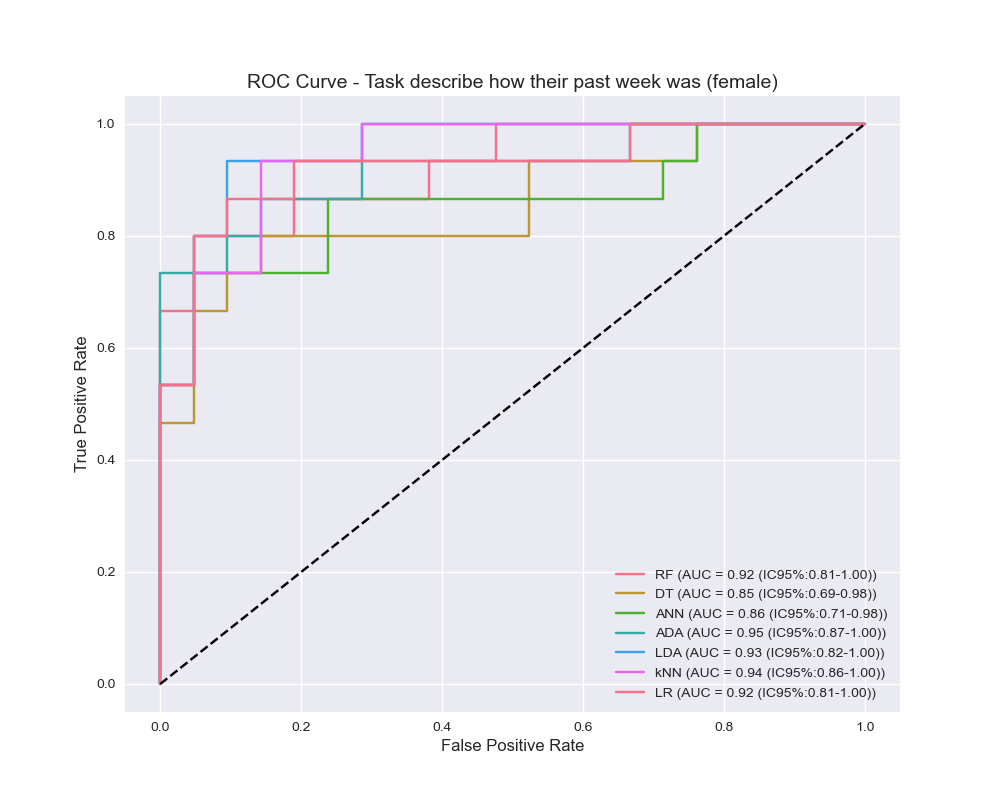
**

**ROC - “Counting from 1 to 10” - Male**

**
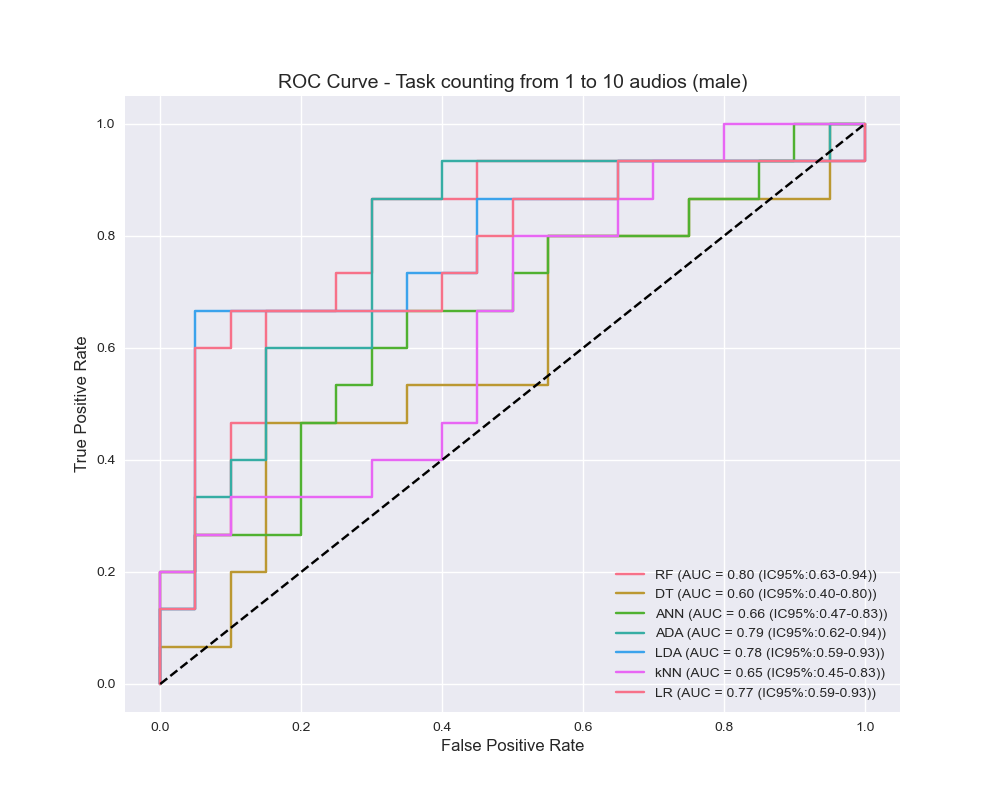
**

**ROC - “How their past week was” - Male**

**
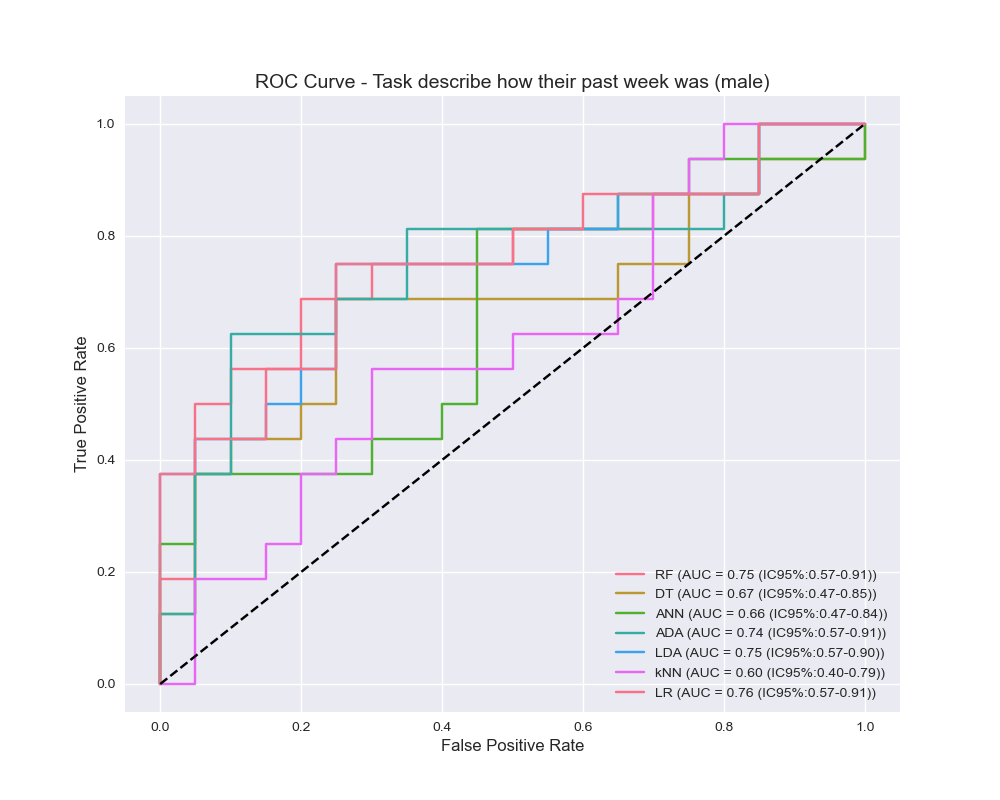
**
