# Supplementary material for: ML-based detection of depressive profile through voice analysis in WhatsApp™ audio messages of Brazilian Portuguese Speakers
Source: PLOS Ment Health. 2026 Jan 21;3(1):e0000357. doi: 10.1371/journal.pmen.0000357 (PMC12822941; doi:10.1371/journal.pmen.0000357)
Supplement: S3 Text — Includes the ROC curve plots for each model and speech task (“Counting from 1 to 10” and “How their past week was”), divided by gender (female and male). These visualizations illustrate model discrimination performance across the different classification settings. (DOCX) [file pmen.0000357.s003.docx]

**Model Performance Tables**

**Task: Describe how their past week was (Female)**

**Main Performance Metrics**

| **Model** | **Recall** | **Specificity** | **PPV** | **NPV** | **Accuracy** | **F1-Score** | **AUC** | **Threshold** |
| --- | --- | --- | --- | --- | --- | --- | --- | --- |
| ADA | 0.894 | 0.908 | 0.892 | 0.928 | 0.900 | 0.881 | 0.945 | 0.591 |
| ANN | 0.793 | 0.922 | 0.892 | 0.863 | 0.867 | 0.829 | 0.860 | 0.600 |
| DT | 0.782 | 0.911 | 0.869 | 0.856 | 0.856 | 0.814 | 0.851 | 0.569 |
| kNN | 0.943 | 0.875 | 0.847 | 0.958 | 0.902 | 0.886 | 0.944 | 0.773 |
| LDA | 0.927 | 0.923 | 0.898 | 0.945 | 0.924 | 0.909 | 0.931 | 0.587 |
| LR | 0.896 | 0.919 | 0.891 | 0.926 | 0.909 | 0.888 | 0.925 | 0.589 |
| RF | 0.840 | 0.941 | 0.922 | 0.894 | 0.898 | 0.871 | 0.925 | 0.622 |

**Confidence Intervals**

| **Model** | **Recall CI** | **Specificity CI** | **PPV CI** | **NPV CI** | **Accuracy CI** | **F1-Score CI** | **AUC CI** |
| --- | --- | --- | --- | --- | --- | --- | --- |
| ADA | (0.688-1.000) | (0.682-1.000) | (0.619-1.000) | (0.773-1.000) | (0.806-0.972) | (0.743-0.976) | (0.869-0.997) |
| ANN | (0.533-1.000) | (0.724-1.000) | (0.625-1.000) | (0.708-1.000) | (0.750-0.972) | (0.667-0.960) | (0.711-0.981) |
| DT | (0.538-1.000) | (0.727-1.000) | (0.600-1.000) | (0.696-1.000) | (0.750-0.972) | (0.636-0.945) | (0.694-0.977) |
| kNN | (0.750-1.000) | (0.667-1.000) | (0.625-1.000) | (0.800-1.000) | (0.806-0.972) | (0.759-0.977) | (0.864-0.996) |
| LDA | (0.769-1.000) | (0.782-1.000) | (0.714-1.000) | (0.818-1.000) | (0.833-1.000) | (0.774-1.000) | (0.819-1.000) |
| LR | (0.706-1.000) | (0.760-1.000) | (0.684-1.000) | (0.800-1.000) | (0.806-1.000) | (0.758-1.000) | (0.812-1.000) |
| RF | (0.615-1.000) | (0.680-1.000) | (0.654-1.000) | (0.750-1.000) | (0.778-0.972) | (0.720-0.973) | (0.815-0.997) |

**Error Rates**

| **Model** | **FPR** | **FNR** | **FPR CI** | **FNR CI** |
| --- | --- | --- | --- | --- |
| ADA | 0.092 | 0.106 | (0.000-0.318) | (0.000-0.312) |
| ANN | 0.078 | 0.207 | (0.000-0.276) | (0.000-0.467) |
| DT | 0.089 | 0.218 | (0.000-0.273) | (0.000-0.462) |
| kNN | 0.125 | 0.057 | (0.000-0.333) | (0.000-0.250) |
| LDA | 0.077 | 0.073 | (0.000-0.218) | (0.000-0.231) |
| LR | 0.081 | 0.104 | (0.000-0.240) | (0.000-0.294) |
| RF | 0.059 | 0.160 | (0.000-0.320) | (0.000-0.385) |

**Model Legend:**

- ADA: AdaBoost
- ANN: Artificial Neural Network
- DT: Decision Tree
- kNN: k-Nearest Neighbors
- LDA: Linear Discriminant Analysis
- LR: Logistic Regression
- RF: Random Forest

**Metrics:**

- CI: Confidence Interval
- PPV: Positive Predictive Value
- NPV: Negative Predictive Value
- AUC: Area Under the Curve
- FPR: False Positive Rate
- FNR: False Negative Rate

**Task: Describe how their past week was (Male)**

**Main Performance Metrics**

| **Model** | **Recall** | **Specificity** | **PPV** | **NPV** | **Accuracy** | **F1-Score** | **AUC** | **Threshold** |
| --- | --- | --- | --- | --- | --- | --- | --- | --- |
| ADA | 0.712 | 0.846 | 0.801 | 0.791 | 0.785 | 0.739 | 0.745 | 0.549 |
| ANN | 0.673 | 0.747 | 0.750 | 0.769 | 0.713 | 0.654 | 0.661 | 0.404 |
| DT | 0.644 | 0.820 | 0.757 | 0.749 | 0.740 | 0.676 | 0.673 | 0.554 |
| kNN | 0.709 | 0.617 | 0.645 | 0.783 | 0.654 | 0.628 | 0.599 | 0.602 |
| LDA | 0.697 | 0.829 | 0.794 | 0.786 | 0.771 | 0.717 | 0.746 | 0.503 |
| LR | 0.705 | 0.832 | 0.794 | 0.790 | 0.775 | 0.723 | 0.758 | 0.498 |
| RF | 0.680 | 0.865 | 0.819 | 0.773 | 0.781 | 0.727 | 0.753 | 0.600 |

**Confidence Intervals**

| **Model** | **Recall CI** | **Specificity CI** | **PPV CI** | **NPV CI** | **Accuracy CI** | **F1-Score CI** | **AUC CI** |
| --- | --- | --- | --- | --- | --- | --- | --- |
| ADA | (0.438-0.941) | (0.591-1.000) | (0.555-1.000) | (0.607-0.952) | (0.667-0.917) | (0.552-0.889) | (0.571-0.910) |
| ANN | (0.250-1.000) | (0.368-1.000) | (0.452-1.000) | (0.533-1.000) | (0.556-0.861) | (0.400-0.837) | (0.475-0.836) |
| DT | (0.312-0.929) | (0.588-1.000) | (0.500-1.000) | (0.552-0.933) | (0.611-0.861) | (0.435-0.850) | (0.466-0.855) |
| kNN | (0.222-1.000) | (0.143-1.000) | (0.394-1.000) | (0.526-1.000) | (0.472-0.806) | (0.364-0.800) | (0.401-0.790) |
| LDA | (0.333-0.951) | (0.591-1.000) | (0.526-1.000) | (0.591-0.959) | (0.639-0.889) | (0.500-0.875) | (0.574-0.898) |
| LR | (0.357-0.947) | (0.571-1.000) | (0.529-1.000) | (0.586-0.955) | (0.666-0.889) | (0.500-0.882) | (0.575-0.909) |
| RF | (0.400-0.933) | (0.640-1.000) | (0.556-1.000) | (0.577-0.941) | (0.639-0.889) | (0.533-0.884) | (0.571-0.909) |

**Error Rates**

| **Model** | **FPR** | **FNR** | **FPR CI** | **FNR CI** |
| --- | --- | --- | --- | --- |
| ADA | 0.154 | 0.288 | (0.000-0.409) | (0.059-0.562) |
| ANN | 0.253 | 0.327 | (0.000-0.632) | (0.000-0.750) |
| DT | 0.180 | 0.356 | (0.000-0.412) | (0.071-0.688) |
| kNN | 0.383 | 0.291 | (0.000-0.857) | (0.000-0.778) |
| LDA | 0.171 | 0.303 | (0.000-0.409) | (0.049-0.667) |
| LR | 0.168 | 0.295 | (0.000-0.429) | (0.053-0.643) |
| RF | 0.135 | 0.320 | (0.000-0.360) | (0.067-0.600) |

**Model Legend:**

- ADA: AdaBoost
- ANN: Artificial Neural Network
- DT: Decision Tree
- kNN: k-Nearest Neighbors
- LDA: Linear Discriminant Analysis
- LR: Logistic Regression
- RF: Random Forest

**Metrics:**

- CI: Confidence Interval
- PPV: Positive Predictive Value
- NPV: Negative Predictive Value
- AUC: Area Under the Curve
- FPR: False Positive Rate
- FNR: False Negative Rate

**Task: Counting from 1 to 10 (Female)**

**Main Performance Metrics**

| **Model** | **Recall** | **Specificity** | **PPV** | **NPV** | **Accuracy** | **F1-Score** | **AUC** | **Threshold** |
| --- | --- | --- | --- | --- | --- | --- | --- | --- |
| ADA | 0.861 | 0.681 | 0.721 | 0.908 | 0.759 | 0.746 | 0.786 | 0.509 |
| ANN | 0.695 | 0.793 | 0.738 | 0.778 | 0.749 | 0.697 | 0.715 | 0.619 |
| DT | 0.717 | 0.839 | 0.809 | 0.809 | 0.785 | 0.736 | 0.786 | 0.620 |
| kNN | 0.929 | 0.790 | 0.777 | 0.937 | 0.850 | 0.840 | 0.875 | 0.777 |
| LDA | 0.780 | 0.826 | 0.797 | 0.841 | 0.806 | 0.769 | 0.828 | 0.578 |
| LR | 0.784 | 0.842 | 0.812 | 0.841 | 0.816 | 0.781 | 0.836 | 0.578 |
| RF | 0.758 | 0.961 | 0.942 | 0.841 | 0.873 | 0.832 | 0.863 | 0.664 |

**Confidence Intervals**

| **Model** | **Recall CI** | **Specificity CI** | **PPV CI** | **NPV CI** | **Accuracy CI** | **F1-Score CI** | **AUC CI** |
| --- | --- | --- | --- | --- | --- | --- | --- |
| ADA | (0.412-1.000) | (0.381-1.000) | (0.458-1.000) | (0.636-1.000) | (0.622-0.892) | (0.560-0.884) | (0.624-0.913) |
| ANN | (0.375-1.000) | (0.440-1.000) | (0.450-1.000) | (0.586-1.000) | (0.595-0.865) | (0.480-0.857) | (0.535-0.870) |
| DT | (0.438-1.000) | (0.450-1.000) | (0.500-1.000) | (0.600-1.000) | (0.649-0.892) | (0.560-0.872) | (0.623-0.918) |
| kNN | (0.733-1.000) | (0.600-1.000) | (0.571-1.000) | (0.769-1.000) | (0.730-0.946) | (0.692-0.952) | (0.743-0.970) |
| LDA | (0.437-1.000) | (0.500-1.000) | (0.533-1.000) | (0.647-1.000) | (0.676-0.919) | (0.592-0.909) | (0.673-0.944) |
| LR | (0.462-1.000) | (0.571-1.000) | (0.550-1.000) | (0.654-1.000) | (0.676-0.919) | (0.600-0.917) | (0.684-0.952) |
| RF | (0.500-1.000) | (0.826-1.000) | (0.750-1.000) | (0.678-1.000) | (0.757-0.973) | (0.667-0.966) | (0.717-0.983) |

**Error Rates**

| **Model** | **FPR** | **FNR** | **FPR CI** | **FNR CI** |
| --- | --- | --- | --- | --- |
| ADA | 0.319 | 0.139 | (0.000-0.619) | (0.000-0.588) |
| ANN | 0.207 | 0.305 | (0.000-0.560) | (0.000-0.625) |
| DT | 0.161 | 0.283 | (0.000-0.550) | (0.000-0.562) |
| kNN | 0.210 | 0.071 | (0.000-0.400) | (0.000-0.267) |
| LDA | 0.174 | 0.220 | (0.000-0.500) | (0.000-0.563) |
| LR | 0.158 | 0.216 | (0.000-0.429) | (0.000-0.538) |
| RF | 0.039 | 0.242 | (0.000-0.174) | (0.000-0.500) |

**Model Legend:**

- ADA: AdaBoost
- ANN: Artificial Neural Network
- DT: Decision Tree
- kNN: k-Nearest Neighbors
- LDA: Linear Discriminant Analysis
- LR: Logistic Regression
- RF: Random Forest

**Metrics:**

- CI: Confidence Interval
- PPV: Positive Predictive Value
- NPV: Negative Predictive Value
- AUC: Area Under the Curve
- FPR: False Positive Rate
- FNR: False Negative Rate

**Task: Counting from 1 to 10 (Male)**

**Main Performance Metrics**

| **Model** | **Recall** | **Specificity** | **PPV** | **NPV** | **Accuracy** | **F1-Score** | **AUC** | **Threshold** |
| --- | --- | --- | --- | --- | --- | --- | --- | --- |
| ADA | 0.880 | 0.727 | 0.715 | 0.897 | 0.793 | 0.778 | 0.787 | 0.490 |
| ANN | 0.650 | 0.747 | 0.705 | 0.754 | 0.704 | 0.637 | 0.660 | 0.395 |
| DT | 0.620 | 0.738 | 0.682 | 0.739 | 0.687 | 0.612 | 0.596 | 0.525 |
| kNN | 0.729 | 0.658 | 0.681 | 0.809 | 0.688 | 0.645 | 0.654 | 0.544 |
| LDA | 0.704 | 0.920 | 0.886 | 0.812 | 0.829 | 0.771 | 0.779 | 0.517 |
| LR | 0.690 | 0.903 | 0.861 | 0.800 | 0.810 | 0.751 | 0.766 | 0.512 |
| RF | 0.846 | 0.767 | 0.744 | 0.875 | 0.801 | 0.780 | 0.796 | 0.558 |

**Confidence Intervals**

| **Model** | **Recall CI** | **Specificity CI** | **PPV CI** | **NPV CI** | **Accuracy CI** | **F1-Score CI** | **AUC CI** |
| --- | --- | --- | --- | --- | --- | --- | --- |
| ADA | (0.583-1.000) | (0.500-0.955) | (0.500-0.941) | (0.706-1.000) | (0.657-0.914) | (0.609-0.909) | (0.618-0.938) |
| ANN | (0.231-1.000) | (0.312-1.000) | (0.407-1.000) | (0.533-1.000) | (0.543-0.829) | (0.375-0.815) | (0.470-0.829) |
| DT | (0.250-1.000) | (0.320-1.000) | (0.400-1.000) | (0.536-1.000) | (0.514-0.829) | (0.375-0.811) | (0.401-0.803) |
| kNN | (0.231-1.000) | (0.238-1.000) | (0.400-1.000) | (0.562-1.000) | (0.514-0.829) | (0.375-0.821) | (0.454-0.826) |
| LDA | (0.444-0.950) | (0.579-1.000) | (0.588-1.000) | (0.636-0.962) | (0.686-0.943) | (0.580-0.917) | (0.587-0.932) |
| LR | (0.429-1.000) | (0.545-1.000) | (0.560-1.000) | (0.625-1.000) | (0.657-0.943) | (0.545-0.909) | (0.587-0.931) |
| RF | (0.600-1.000) | (0.520-1.000) | (0.500-1.000) | (0.684-1.000) | (0.657-0.914) | (0.609-0.909) | (0.632-0.942) |

**Error Rates**

| **Model** | **FPR** | **FNR** | **FPR CI** | **FNR CI** |
| --- | --- | --- | --- | --- |
| ADA | 0.273 | 0.120 | (0.045-0.500) | (0.000-0.417) |
| ANN | 0.253 | 0.350 | (0.000-0.688) | (0.000-0.769) |
| DT | 0.262 | 0.380 | (0.000-0.680) | (0.000-0.750) |
| kNN | 0.342 | 0.271 | (0.000-0.762) | (0.000-0.769) |
| LDA | 0.080 | 0.296 | (0.000-0.421) | (0.050-0.556) |
| LR | 0.097 | 0.310 | (0.000-0.455) | (0.000-0.571) |
| RF | 0.233 | 0.154 | (0.000-0.480) | (0.000-0.400) |

**Model Legend:**

- ADA: AdaBoost
- ANN: Artificial Neural Network
- DT: Decision Tree
- kNN: k-Nearest Neighbors
- LDA: Linear Discriminant Analysis
- LR: Logistic Regression
- RF: Random Forest

**Metrics:**

- CI: Confidence Interval
- PPV: Positive Predictive Value
- NPV: Negative Predictive Value
- AUC: Area Under the Curve
- FPR: False Positive Rate
- FNR: False Negative Rate
