# Supplementary material for: ML-based detection of depressive profile through voice analysis in WhatsApp™ audio messages of Brazilian Portuguese Speakers
Source: PLOS Ment Health. 2026 Jan 21;3(1):e0000357. doi: 10.1371/journal.pmen.0000357 (PMC12822941; doi:10.1371/journal.pmen.0000357)
Supplement: S4 Text — Presents absolute and normalized confusion matrices for each classification model, gender, and speech task. These matrices illustrate prediction distributions and support interpretation of model precision and recall performance. (DOCX) [file pmen.0000357.s004.docx]

Classification by AUC and statistical significance

Task: how their past week was (Female)

Model p_value AUC Cohen_d Diff_% Status

Media ADA 0.000004 0.946032 1.989493 19.450262 Significant

Media kNN 0.000004 0.942857 1.373486 12.790889 Significant

Media LDA 0.000007 0.930159 1.795997 25.035201 Significant

Media LR 0.000010 0.923810 1.803347 24.871484 Significant

Media RF 0.000010 0.923810 2.079602 19.062011 Significant

Media ANN 0.000144 0.860317 1.366099 18.114308 Significant

Media DT 0.000184 0.853968 1.585286 13.264572 Significant

Task: how their past week was (Male)

Model p_value AUC Cohen_d Diff_% Status

Media LR 0.004735 0.756250 0.937111 14.235832 Significant

Media RF 0.005192 0.753125 0.988169 10.014622 Significant

Media ADA 0.006225 0.746875 0.904829 9.279111 Significant

Media LDA 0.006225 0.746875 0.917039 14.291697 Significant

Media DT 0.041366 0.671875 0.591295 4.824472 Significant

Media ANN 0.053950 0.659375 0.635787 10.112714 *Marginal*

Media kNN 0.150414 0.603125 0.353260 3.348800 *Not Significant*

Task: counting from 1 to 10 (Female)

Model p_value AUC Cohen_d Diff_% Status

Media kNN 0.000068 0.872024 1.243084 10.531994 Significant

Media RF 0.000098 0.863095 1.394690 16.268256 Significant

Media LR 0.000353 0.830357 1.329884 19.508774 Significant

Media LDA 0.000353 0.830357 1.317559 19.690536 Significant

Media DT 0.001546 0.788690 1.129220 12.154526 Significant

Media ADA 0.001707 0.785714 1.226379 12.814632 Significant

Media ANN 0.013120 0.717262 0.685025 9.530185 Significant

Task: counting from 1 to 10 (Male)

Model p_value AUC Cohen_d Diff_% Status

Media RF 0.001769 0.793333 0.947785 9.464202 Significant

Media ADA 0.002426 0.783333 0.978299 10.959246 Significant

Media LDA 0.002690 0.780000 0.945583 14.700276 Significant

Media LR 0.003645 0.770000 0.944061 14.493084 Significant

Media ANN 0.056673 0.660000 0.612935 8.088466 Marginal

Media kNN 0.078290 0.643333 0.580916 6.430308 Marginal

Media DT 0.162705 0.600000 0.292592 2.886249 Not Significant

============================================================

LEGEND:

✅ SIGNIFICANT: p < 0.05

⚠️ MARGINALLY SIGNIFICANT: p < 0.10

❌ NOT SIGNIFICANT: p ≥ 0.10

Cohen's d: 0.2=small, 0.5=medium, 0.8=large

AUC: 0.5=random, 0.7=good, 0.8=very good, 0.9=excellent
