# Supplementary material for: ML-based detection of depressive profile through voice analysis in WhatsApp™ audio messages of Brazilian Portuguese Speakers
Source: PLOS Ment Health. 2026 Jan 21;3(1):e0000357. doi: 10.1371/journal.pmen.0000357 (PMC12822941; doi:10.1371/journal.pmen.0000357)
Supplement: Attachment — Submitted filename: RL_DepressionVoice.docx [file pmen.0000357.s005.docx]

**Prof. Lucas Murrins Marques, PhD**

Mental Health Department, Santa Casa de São Paulo School of Medical Sciences, São Paulo, SP, Brazil.

Dona Veridiana Street 55, 3o. Floor, Higienópolis – São Paulo / SP, Brazil

ORCID: 0000-0003-2999-2448

Email: [lucas.murrins@fcmsantacasasp.edu.br](mailto:lucas.murrins@fcmsantacasasp.edu.br)

**ML-Based Detection of Depressive Profile Through Voice Analysis in WhatsApp™ Audio Messages of Brazilian Portuguese Speakers**

Dear Editorial team,

We thank the Editor and Reviewers for their thorough and constructive feedback on our manuscript titled "*ML-Based Detection of Depressive Profile Through Voice Analysis in WhatsApp™ Audio Messages of Brazilian Portuguese Speakers*". We have carefully revised the manuscript to address each of the comments provided.

In this rebuttal letter, we respond to all points raised by the Editor and Reviewers in detail. For clarity, all changes made in the manuscript have been highlighted in yellow, following the journal's formatting guidelines.

We appreciate the opportunity to improve our manuscript and believe that the current version has benefited significantly from the insightful suggestions provided. Below, we provide a point-by-point response to each comment.

**Sincerely,**

**Lucas Murrins Marques**

______________________________________________________________________

**Editorial comments:**

1) Thank you for submitting your manuscript to PLOS Mental Health. After careful consideration, we feel that it has merit but does not fully meet PLOS Mental Health’s publication criteria as it currently stands. Therefore, we invite you to submit a revised version of the manuscript that addresses the points raised during the review process.

*R: We sincerely thank the editor and reviewers for their careful evaluation of our manuscript and for providing constructive feedback. We have thoroughly revised the manuscript to address all the points raised during the review process. These revisions include additional analyses, methodological clarifications, expanded discussions, and improvements in figures, tables, and language. We believe that these changes have substantially strengthened the manuscript and enhanced its clarity, robustness, and contribution to the field.*

______________________________________________________________________

**Reviewers' comments:**

**Reviewer #1:**

Manuscript Summary:

1) This study leverages WhatsApp voice messages from Brazilian Portuguese speakers to detect depression with traditional ML models. The real-world data scenario is commendable and the methodology is generally sound. However, key shortcomings such as sample imbalance, insufficient statistical testing, and limited interpretability must be thoroughly addressed before publication. And the combination of acoustic features and machine learning for depression detection has been extensively explored; this study does not adopt newer architectures (e.g., Transformers) nor does it benchmark results on additional datasets such as AVEC:

*R: We thank the reviewer for recognizing the strengths of our work and for the constructive feedback. All major concerns have been addressed in the revised manuscript: we maintained the real-world gender distribution but reported and interpreted results separately for men and women, adding discussion on the need for balanced recruitment in future studies; improved statistical rigor by including 95% confidence intervals, and detailed confusion matrices with FPR/FNR and misclassification examples (full outputs in Supplementary Material); clarified the controlled-access process for dataset/code sharing; expanded discussion on domain shift, device variability, biological relevance of key features (e.g., MFCC2), and possible medication confounding (with an ongoing study to address this); strengthened the Ethics section with details on voice de-identification, secure storage, and controlled access; unified terminology (“sample” vs. “frame”); and refined Figures 3/4 for clarity. These revisions collectively enhance statistical robustness, interpretability, and transparency while preserving the ecological validity of our approach.*

Major Concerns:

1) Increase the male sample size or apply resampling/re-weighting to mitigate gender imbalance.N:

*R: We appreciate the reviewer’s important observation regarding gender imbalance. As our study was designed to capture real-world, naturalistic voice samples from patients and healthy volunteers, recruitment was subject to the gender distribution of individuals available in our clinical and community settings, which led to a higher proportion of female participants. Rather than artificially balancing the dataset or applying re-weighting — which could have altered the ecological validity of the findings — we chose to present and interpret model performance separately for men and women. This approach allows us to transparently assess how gender distribution may affect classification accuracy. Our goal in this initial study was not to develop a fully gender-balanced AI model, but to explore the feasibility of WhatsApp™-based depression detection in real-world conditions. We have now explicitly discussed this rationale and its implications in the “Limitations” section, highlighting the need for future studies with balanced recruitment strategies to further validate cross-gender performance.*

2) Add confidence intervals and hypothesis tests (e.g., significance of AUC and accuracy differences).

*R: We thank the reviewer for this valuable suggestion. In the revised manuscript, we now report 95% confidence intervals for all key performance metrics (accuracy, AUC, sensitivity, specificity, PPV, NPV, and F1-score), estimated via bootstrap with 1,000 iterations stratified by sex. These results are presented in the Results section and summarized in Table 9, with full details provided in Supplementary 3. Confusion matrices (absolute and normalized) remain available in Supplementary 1, and ROC curves for each task and gender combination are included in Supplementary 2. Together, these additions enhance statistical robustness and transparency of our findings.*

3) Provide confusion matrices, FPR/FNR, and representative misclassification analyses to enhance transparency.

*R: We thank the reviewer for this important comment. As mentioned in our previous response, we have provided detailed confusion matrices (absolute and normalized) for each task, gender, and best-performing model in the Supplementary Material (Supplementary 1). From these matrices, we calculated and report the false positive rate (FPR) and false negative rate (FNR) for each condition. In addition, we have included representative misclassification examples in the Results section to illustrate the nature of some errors, such as misclassifications potentially related to background noise or neutral/monotone speech delivery. Together, these additions enhance the transparency and interpretability of our results.*

4) Recommend releasing anonymized code and acoustic-feature datasets to ensure reproducibility.

*R: We appreciate the reviewer’s suggestion regarding reproducibility. All data used in this study were anonymized to ensure participant privacy and comply with ethical standards. As stated in the revised manuscript, both the processed acoustic-feature datasets and the machine learning model code are intellectual property of the company where the research was developed, and thus cannot be openly released. However, in line with our Data Availability Statement, these materials may be shared with qualified researchers upon reasonable request to the corresponding author, contingent on company approval and compliance with ethical and privacy regulations. This controlled-access approach ensures adherence to data protection principles while enabling scientific verification and potential collaboration.*

5) Recommend adding validation experiments on additional publicly available datasets (e.g., AVEC, DAIC-WOZ) to demonstrate cross-corpus generalizability and strengthen the robustness of the findings.

*R: We thank the reviewer for this valuable suggestion. We fully agree that evaluating cross-corpus generalizability is an important step toward validating and strengthening the robustness of voice-based depression detection models. Our research group is currently conducting a follow-up study using additional publicly available datasets such as AVEC and DAIC-WOZ, including data in other languages, to assess performance across different corpora and recording conditions. These analyses will be reported in a forthcoming publication. In the present study, our primary objective was to develop and validate a machine learning model trained and tested on real-world, naturalistic WhatsApp™ audio data from Brazilian Portuguese speakers, and the conclusions are therefore bounded to this scope. Nonetheless, we recognize the value of such external validation and have added a note in the Discussion to highlight this as an important direction for future work.*

6) Discuss potential domain-shift effects between training (spontaneous speech) and testing (structured tasks).

*R: We appreciate the reviewer’s observation regarding the domain shift between spontaneous speech in the training set and structured tasks in the testing set. This design choice was intentional, aiming to simulate real-world clinical scenarios in which training data may come from unstructured patient communications while screening or monitoring tasks may involve semi-structured speech. We agree that such differences can affect model performance, potentially introducing a bias toward distinguishing speech styles rather than depressive states. We have expanded the Discussion to address this point, highlighting it as both a limitation and a potential advantage, since it allowed us to assess the model’s ability to generalize across task types. Furthermore, we note that this domain-shift evaluation aligns with our broader goal of testing the model under varied, ecologically valid conditions, while also motivating future work with matched-speech validation to isolate the effect of speech style.*

7) Clarify how recording devices, ambient noise, and microphone variability were documented or controlled.

*R: We appreciate the reviewer’s question regarding recording devices, ambient noise, and microphone variability. In this study, we intentionally did not control for smartphone models or microphone types, as our primary objective was to evaluate model performance under ecologically valid, real-world conditions that reflect actual clinical and telehealth scenarios. Participants used their own mobile devices in their natural environments, which inherently introduced variability in audio quality and background noise. We did document the general type of device (e.g., Android vs. iOS) when available, although this information was not used as a covariate in the present analysis. To mitigate the potential impact of recording artifacts, all audio underwent standardized pre-processing, including silence removal and noise filtering, as described in the Methods section. We have clarified this rationale and these procedures in the revised manuscript and added a note in the Limitations acknowledging that microphone and device variability may influence acoustic features and classification performance.*

8) Explain the biological relevance of key features (e.g., MFCC2) to depressive pathophysiology.

*R: We thank the reviewer for this important observation. In the revised manuscript, we have expanded the Discussion to include a detailed explanation of the potential biological relevance of the key acoustic features identified by our models, particularly MFCC2, which emerged as the most important feature in several classifiers. MFCC2 captures spectral envelope information related to the shape and dynamics of the vocal tract during speech production, which can be influenced by respiratory control, laryngeal muscle tension, and articulatory precision. Depression has been associated with psychomotor slowing, reduced prosody, and alterations in respiratory-laryngeal coordination, which may manifest as shifts in formant structure and spectral energy distribution — changes that MFCC2 is sensitive to. We have also added context on how other high-ranking features, such as spectral rolloff and zero-crossing rate, may reflect reduced high-frequency energy and flatter pitch contours, both of which are frequently reported in depressive speech profiles.*

9) Add sensitivity analyses or discussion on confounders such as medications.

*R: We thank the reviewer for this important point. In the recruitment process, we excluded participants with major medical or neurological conditions, current smoking, anabolic steroid use, and recent respiratory infections or allergies, in order to reduce physiological confounders that could affect voice. While we did not exclude participants on stable psychiatric medication, we recognize that medication effects on vocal characteristics may influence acoustic features. In the revised manuscript, we have expanded the Discussion to explicitly address this limitation. We are also conducting a separate study, led by a graduate student, specifically designed to investigate the impact of medication-related confounders on our models. The results of that work will be reported in a forthcoming publication, allowing a deeper understanding of how pharmacological treatments interact with voice-based depression detection.*

10) Expand the ethics statement to detail voice de-identification, data storage, and access protocols.

*R: We appreciate the reviewer’s suggestion. We have expanded the ethics section to include detailed information on voice data de-identification, secure storage, and controlled access procedures. Specifically, all voice recordings were de-identified at the point of collection, assigned unique alphanumeric codes, and stripped of any personally identifiable metadata. Data were stored on secure, access-controlled institutional servers with encryption in transit and at rest, with raw audio access restricted to the core research team. Only anonymized acoustic feature datasets were used for analysis. These additions have been incorporated into the Design section of the manuscript.*

11) Edit language for conciseness and unify terminology (e.g., “frame” vs. “sample”).

*R: We have revised the manuscript to explicitly define and consistently use the terms sample and frame throughout the text. In our study, sample refers to the complete audio file obtained from each participant (e.g., a single WhatsApp message), while frame refers to a short, fixed-duration segment of that sample generated during the feature extraction process. This clarification has been incorporated into the Audio Signal Preprocessing section (Methods) and applied consistently across the manuscript to avoid ambiguity and ensure methodological clarity.*

12) Refine Figures 3/4: clarify y-axis labels and legends to avoid ambiguity.

*R: We revised Figures 3 and 4 to improve clarity and avoid ambiguity. The y-axis label was updated to explicitly read “Proportion of audio frames classified as depressive (0.0 = no frames classified as depressive; 1.0 = all frames classified as depressive)”, ensuring consistency with the terminology used throughout the manuscript. The legend wording was refined to consistently use “frames” when referring to segmented portions of each audio sample, and to explicitly state that the dashed red line represents the classification threshold, where values above this threshold indicate a “depressive” classification at the frame level. Additionally, all abbreviations for machine learning models (ADA, RF, DT, kNN, LR, LDA, ANN) are now defined in the figure captions to facilitate reader understanding.*

______________________________________________________________________

**Reviewer #2:**

Manuscript Summary:

1) This manuscript presents a study on machine learning (ML)-based detection of depressive profiles using voice analysis from WhatsApp™ audio messages in Brazilian Portuguese speakers. The work addresses an important clinical need and demonstrates methodological rigor in several respects. However, there are a few areas where the statistical and methodological approach could be strengthened. Below are several suggestions to help improve the manuscript:

*R: We appreciate the reviewer’s observation regarding the domain shift between spontaneous speech in training and structured speech in testing. This choice was intentional to simulate realistic clinical and telehealth scenarios where models trained on unstructured communications may be applied in semi-structured screenings. While dataset size constraints precluded matched-speech or ablation analyses, we acknowledge this as a limitation and have expanded the Discussion accordingly. Related issues raised in other comments—such as gender performance differences (Supplementary1.pdf, Supplementary2.pdf), diagnostic criteria differences (DSM-5 vs. MINI), and feature stability (MFCC2 as the only consistent top feature)—are now clarified in the manuscript. We also emphasize that working with uncontrolled, real-world audio conditions was a deliberate strategy to enhance ecological validity and generalizability.*

Major Concerns:

1) The study uses different speech types for training (spontaneous) and testing (structured), introducing a domain shift that may bias the model toward distinguishing speech styles rather than depressive states. It would enhance the study’s validity to evaluate performance within matched speech types, conduct ablation studies with mixed and separated datasets, and assess whether the key features identified (e.g., in Table 7) are consistent across validation schemes.

*R: We appreciate the reviewer’s important observation regarding the domain shift between spontaneous speech in training and structured speech in testing. This design choice was intentional, aiming to simulate realistic clinical and telehealth scenarios in which models trained on unstructured patient communications may later be applied in semi-structured screening contexts. We agree that such differences can influence performance and potentially bias models toward detecting speech-style differences rather than depressive states. While the present study did not include matched-speech or ablation analyses due to dataset size constraints, we acknowledge this as a methodological limitation and have expanded the Discussion to address its implications. We also note that, in follow-up work currently underway, we are evaluating models within matched-speech conditions and across mixed/segregated datasets, as well as comparing the stability of key features such as MFCC2 across validation schemes. These future results will allow a more granular assessment of generalization and feature robustness across speech contexts.*

2) The reported model performance differs notably by gender (91.67% accuracy for women vs. 80% for men), but the male sample sizes are quite limited (8 in training, 16 in testing). It would be helpful to report confidence intervals, test the significance of these gender differences, and perform a power analysis. If the sample size is underpowered for subgroup analysis, the authors should consider reporting pooled results and clearly state the limitations.

*R: We appreciate this insightful comment regarding gender differences in model performance. In response, we have conducted additional analyses to explore model performance stratified by gender, including ROC curves for each task and gender combination, as well as absolute and normalized confusion matrices. These results are now provided in the supplementary materials: Supplementary1.pdf contains the confusion matrices, and Supplementary2.pdf presents the ROC curves for both the “Counting from 1 to 10” and “How their past week was” tasks, separately for male and female participants. While these analyses illustrate the observed differences, we note that the male subgroup size remains limited (n=8 in training, n=16 in testing), and as such, these results should be interpreted cautiously. Given the small sample size for men, we have opted to maintain the pooled analysis as the primary result and to explicitly state in the revised manuscript that subgroup analyses by gender are underpowered and exploratory. No major modifications to the main text are required beyond referencing these supplementary materials and clarifying this limitation in the Discussion section.*

3) The training and testing groups differ in diagnostic criteria: DSM-5-based clinical evaluations were used for the training set, while the MINI interview was used for the test set. This inconsistency raises concerns about the comparability and validity of the diagnostic labels. Clarifying these differences and discussing their implications would strengthen the clinical foundation of the study.

*R: We thank the reviewer for this important observation. In our study, the diagnostic criteria for the training and testing sets were indeed based on different structured clinical assessments — DSM-5-based evaluations performed by psychiatrists and psychiatry residents for the training set, and the MINI (Mini International Neuropsychiatric Interview) for the testing set. This was primarily due to the fact that these datasets were collected in different clinical contexts and by different teams prior to integration in this study. Both instruments are well-validated for the diagnosis of Major Depressive Disorder and have high concordance rates, but we acknowledge that differences in structure, depth of exploration, and interviewer training could introduce heterogeneity in diagnostic labeling. We have now clarified this methodological point in the Methods section and have added a note in the Limitations highlighting that such differences may have contributed to variability in model performance and should be harmonized in future prospective studies.*

4) Table 7 shows considerable variation in the top 10 acoustic features across models, with only MFCC 2 appearing consistently. This variability raises concerns about feature stability and interpretability. It would be valuable to analyze whether the most important features remain consistent across validation folds and datasets.

*R: We appreciate the reviewer’s observation regarding feature stability. To address this, we examined the top 10 features across validation folds for each model in the training dataset, as well as their consistency when applied to the external test dataset. MFCC 2 was indeed the only feature consistently ranked among the top 10 across all folds and models, confirming its robustness as a potential biomarker. Other features, such as spectral rolloff and zero-crossing rate, appeared recurrently but not uniformly across folds, which is expected given inter-individual variability, noise in real-world recordings, and differences in speech tasks between training and test sets. This variability highlights the complexity of depression-related acoustic patterns and reinforces the need for multi-feature approaches rather than reliance on a single parameter. We have added a statement in the Discussion to clarify this point.*

5) While the paper highlights the practical potential of WhatsApp-based depression screening, several limitations remain underexplored. These include the study’s restriction to uncontrolled audio environments, and the observed gender disparities in performance. The authors are encouraged to discuss plans for handling diverse recording conditions, integrating the tool into real-world healthcare workflows, and addressing ethical considerations such as false positives, privacy, and informed consent.

*R: We appreciate the reviewer’s comment and agree that diverse recording conditions pose challenges for model performance. However, in the present study, this variability was an intentional design choice rather than an uncontrolled limitation. Our aim was to evaluate the feasibility of detecting depressive profiles from WhatsApp™ audio messages under real-world conditions, where background noise, different devices, and variable microphone quality are the norm. This approach enhances ecological validity and increases the likelihood that the models will generalize to practical, large-scale applications outside of controlled laboratory settings. We have clarified this rationale in the Discussion and, while recognizing its implications, we also outline in the revised text our plans to further optimize future models through adaptive noise-handling techniques, task-specific calibration, and integration into healthcare workflows, alongside ethical safeguards.*

______________________________________________________________________
