# Supplementary material for: ML-based detection of depressive profile through voice analysis in WhatsApp™ audio messages of Brazilian Portuguese Speakers
Source: PLOS Ment Health. 2026 Jan 21;3(1):e0000357. doi: 10.1371/journal.pmen.0000357 (PMC12822941; doi:10.1371/journal.pmen.0000357)
Supplement: Attachment — Submitted filename: RL_DepressionVoice_20october.docx [file pmen.0000357.s006.docx]

**Prof. Lucas Murrins Marques, PhD**

Mental Health Department, Santa Casa de São Paulo School of Medical Sciences, São Paulo, SP, Brazil.

Dona Veridiana Street 55, 3o. Floor, Higienópolis – São Paulo / SP, Brazil

ORCID: 0000-0003-2999-2448

Email: [lucas.murrins@fcmsantacasasp.edu.br](mailto:lucas.murrins@fcmsantacasasp.edu.br)

**ML-Based Detection of Depressive Profile Through Voice Analysis in WhatsApp™ Audio Messages of Brazilian Portuguese Speakers**

Dear Editorial team,

We thank the Editor and Reviewers for their thorough and constructive feedback on our manuscript titled "*ML-Based Detection of Depressive Profile Through Voice Analysis in WhatsApp™ Audio Messages of Brazilian Portuguese Speakers*". We have carefully revised the manuscript to address each of the comments provided.

In this rebuttal letter, we respond to all points raised by the Editor and Reviewers in detail. For clarity, all changes made in the manuscript have been highlighted in yellow, following the journal's formatting guidelines.

We appreciate the opportunity to improve our manuscript and believe that the current version has benefited significantly from the insightful suggestions provided. Below, we provide a point-by-point response to each comment.

**Sincerely,**

**Lucas Murrins Marques**

______________________________________________________________________

**Reviewers' comments:**

**Reviewer #3:**

Manuscript Summary:

1) In this work, Authors evaluate several different ML models’ ability to classify depressed patients. The dataset is represented by several WhatsApp audio recordings, from which they extract numeorus audio features. The problem of cheap and objective depression detection and evaluation tools is important nowadays, and the Machine Learning methodlogy seems robust.:

*R: We sincerely thank the reviewer for their thoughtful summary and positive assessment of our work. We truly appreciate the acknowledgment of both the importance of developing accessible and objective tools for depression detection and the robustness of our methodological approach. Our goal in this study was to take a concrete step toward translating affective computing and clinical psychiatry into real-world, low-cost, and scalable digital health solutions, particularly relevant in resource-limited settings.*

*We are grateful for the reviewer’s engagement and constructive feedback, which have significantly contributed to improving the clarity and depth of our revised manuscript.*

Major Concerns:

1) While detecting the presence of depression is surely an important task, as far as I know depression has several degrees. I wonder if reducing it to a dicotomic variable could be an oversimplification and, at the bottom line, present limited clinical benefits. Lines 69-71 seem to underline the same limitation: “[referred to tools used nowadays] Although these tools provide valuable insights into the presence and severity of depression, they often lack the specificity required for clinical phenotyping and individualized treatment strategies”. Does the models you provide have the specificity required for clinical phenotyping? Or are they to be intended as “pre-screening” tools?:

*R: We thank the reviewer for this insightful observation. In this study, we deliberately modeled depression as a binary classification problem (Major Depressive Disorder vs. healthy control) to establish the feasibility of machine learning models in distinguishing depressive from non-depressive voice patterns in naturalistic WhatsApp™ recordings. At this stage, our primary goal was to validate the technical and ecological viability of such models under real-world conditions, rather than to capture the dimensional severity or subtyping of depression.*

*We fully agree that symptom severity and clinical subtyping are essential for translational and personalized applications. Accordingly, we have clarified in both the Introduction and Discussion sections that the current models should be interpreted as pre-screening tools rather than full diagnostic instruments. We have also included a forward-looking statement outlining our plan to extend the present framework toward:*

*(i) ordinal or multiclass classification tasks (e.g., mild, moderate, and severe depression); and*

*(ii) regression-based models predicting continuous scores on validated scales such as the MADRS or PHQ-9.*

*These additions clarify the conceptual scope of the current work and situate it within a broader developmental roadmap for voice-based biomarkers of depression.*

2) *Another major issue is the fact that performance on Test set for female subjects are sensibly higher than Train set and Test set for male subjects. For example, with kNN model:*

*i). Train set F1 = 75%, AUC = 69%*

*ii). Test set on the past-week task, F1 = 84% female (+9% on train set) / 56% male (-19%), AUC = 84 female (+15%) / 60 male (-9%)*

*iii). Test set on the count1-10 task, F1 = 81% female (+6% on train set) / 65% male (-10%), AUC = 83 female (+14%) / 65 male (-4%)*.

*R: We thank the reviewer for this important observation regarding gender-related performance differences and apparent discrepancies between training and test accuracies.*

*The higher accuracy observed for female participants is primarily explained by the sample imbalance, as the dataset contained significantly fewer male samples in the training set. This imbalance likely increased the variance of model estimates for men, resulting in wider confidence intervals, as detailed in Table 9. Rather than artificially balancing the dataset, we chose to preserve the naturalistic gender distribution to maintain ecological validity — a methodological decision now explicitly stated in the Limitations section.*

*Regarding the slightly higher test performance compared to training, this difference stems from how accuracy metrics were computed across stages. The training phase evaluates performance at the frame level (classification of individual audio segments as depressive vs. non-depressive), while the test phase aggregates these frame-level probabilities at the audio level (i.e., proportion of depressive frames per participant audio). Consequently, frame-level noise and misclassifications are averaged in the test phase, yielding higher apparent performance. This rationale is now clarified in the Results section (see revised text around lines 289–290 and 311–313).*

*Together, these clarifications reinforce the interpretability of the results while maintaining transparency regarding gender imbalance and metric-level differences between training and testing.*

3) 3. In my opinion, concerns presented at previous point could have several possible explanations:

i). Test set subjects were evaluated with MINI interview, differently from train subjects (lines 150-151: “structured psychiatric evaluation conducted by experienced psychiatrists in private clinical settings”);

ii). Different content of speeches between Train and test set

iii). Average ages look quite different between Train and Test set, expecially for female sample (36 for Train, 47 for Test). Have you evaluated this difference, other than the intra-group one (e.g., Test male MDD vs Test male HV)?

iv). I would exclude data leakage since the ML methodology you provide seem robust.

*R: We thank the reviewer for this thorough analysis and for recognizing the robustness of our methodology in avoiding data leakage. We agree that the observed differences in performance between training and test datasets may partly reflect variations in diagnostic instruments, speech content, and demographic composition.*

*(i) Diagnostic instruments: The training dataset employed DSM-5–based clinical evaluations conducted by psychiatrists in private settings, whereas the test dataset used the MINI structured interview. Although both are validated tools with high concordance for Major Depressive Disorder diagnosis, subtle differences in structure, interviewer expertise, and contextual phrasing may introduce labeling heterogeneity. We have now clarified this distinction in the Methods section and acknowledged its potential effect in the Limitations.*

*(ii) Speech content: The training and test datasets included spontaneous and semi-structured speech tasks, respectively, intentionally reflecting real-world variability in telehealth and clinical screening contexts. We have expanded the Discussion to explain that while this design allowed us to assess domain generalization, it may also have contributed to cross-corpus performance differences.*

*(iii) Demographic variation: We acknowledge that the test set included slightly older participants, particularly among women (mean age 47 vs. 36 years in the training set). While intra-group analyses (e.g., male MDD vs. male HV) indicated consistent within-group trends, we have added this age difference to the Limitations and will harmonize demographic variables in future datasets.*

*To improve future generalization, we plan to harmonize diagnostic protocols, speech content, and demographic distributions in ongoing data collections, ensuring greater comparability across corpora. These clarifications have been incorporated into the revised manuscript.*

4) 4. I would suggest to harmonize MDD evaluation and content of audio recordings to provide compatible datasets.

*R: We thank the reviewer for this constructive suggestion. We agree that harmonizing diagnostic instruments and speech content across datasets would improve cross-corpus comparability and facilitate stronger generalization. In the present study, our choice to use distinct diagnostic procedures (DSM-5–based vs. MINI) and heterogeneous speech tasks (spontaneous vs. semi-structured) was intentional, aiming to evaluate model performance under ecologically valid and domain-shifted conditions. This approach allowed us to test the feasibility of detecting depressive vocal patterns despite natural variations in recording context and diagnostic workflow, which are common in real-world telehealth and clinical environments.*

*Nonetheless, we fully acknowledge that these methodological differences may introduce mild variability between datasets. Accordingly, we have included in the Discussion and Limitations sections a clear statement outlining our ongoing plan to harmonize (i) diagnostic assessments, (ii) speech-task content, and (iii) demographic distributions in upcoming data collections. These steps will ensure dataset compatibility and enable more rigorous cross-dataset validation in future work.*

5) 5. Still related to point 3, partial explanation of why performance for male sample is sensibly lower than femal sample is provided in lines 411-413. How would you motivate that? Are you suggesting that “traditional masculine norms” modify acoustic features as well? If the answer is yes, this is an interesting point of discussion. If the answer is no, I would expect an acoustic features-based model to be less sensible to such “norms”, as this could be a major limitation.

*R: We thank the reviewer for raising this thought-provoking point. We agree that social and cultural factors — including traditional masculine norms — can influence emotional expressivity and, consequently, affect vocal acoustic features. Research in affective and social neuroscience suggests that men who adhere more strongly to conventional masculine norms often display reduced prosodic variability, lower pitch modulation, and flatter affective tone, particularly in contexts involving self-disclosure or emotional expression. These behavioral tendencies could manifest as attenuated acoustic cues, making depressive speech patterns less distinguishable from neutral speech in male participants.*

*Thus, our interpretation is that these sociocultural influences may interact with biological and emotional regulation processes, leading to subtler acoustic signatures of depression among men. This possibility has been incorporated into the Discussion to contextualize the observed gender-related differences and to emphasize that the model’s performance reflects not only physiological markers but also culturally mediated expression patterns.*

*We have also noted that future work will address this issue by collecting larger, gender-balanced samples and by explicitly modeling social and expressive variability in voice-based biomarkers of depression.*

Minor Concerns:

1) Line 262: please, indicate a reference for nested-CV.

*R: We thank the reviewer for this observation. We have now added appropriate references to support the use of nested cross-validation for model evaluation and hyperparameter optimization. Specifically, we cite Varma and Simon (2006) and Vabalas et al. (2019), both of which discuss the rationale and reliability of nested-CV in preventing information leakage and ensuring unbiased performance estimation. The revised sentence now reads: “Model hyperparameters were optimized using a nested cross-validation scheme (Vabalas et al., 2019).”.*

2) Figure 3 and 4 are hardly readable, please use high-resolution pictures.

*R: Updated.*

3) Line 356: what you developed are classifiers, not predictors.

*R: We thank the reviewer for this correction. We have replaced the term “predictors” with “classifiers” throughout the manuscript to accurately reflect the nature of our models, which perform categorical classification (depressive vs. non-depressive) rather than continuous prediction. This terminology adjustment ensures conceptual and methodological precision.*

4) In Discussion, lines 366-376 are a repetition of what you described in Methods: please remove them.

*R: Removed.*

5) In Discussion, lines 452-473 present interesting data and facts. I would suggest to move them in the intro, to motivate the rationale of the work (see also comment 1).

*R: We thank the reviewer for this valuable suggestion. We agree that these paragraphs provide important epidemiological and societal context and are better suited to the Introduction, where they can strengthen the rationale for the study. We have therefore relocated this section to the beginning of the Introduction, slightly revising its tone to emphasize the public health relevance of depression, suicide prevention, and the potential of mobile and voice-based technologies for early detection and scalable intervention.*

6) In Limitations, lines 490-495 describe a strength of the study. I would suggest to move in the Discussion.

*R: We thank the reviewer for this thoughtful observation. We agree that this aspect—specifically, the intentional use of heterogeneous and ecologically valid voice samples—can indeed be interpreted as a strength of the study. However, we have chosen to retain it in the Limitations section to emphasize the dual nature of this feature: while methodological heterogeneity may constrain strict cross-dataset comparability, it simultaneously enhances ecological validity, real-world applicability, and model generalization.*

*By keeping it in the Limitations, we aim to transparently acknowledge the trade-off between experimental control and external validity, a balance that is central to the study’s design rationale. To clarify this point, we have slightly revised the paragraph to highlight that this methodological choice is both a limitation and a deliberate strength of the approach..*

7) Table 6 and 8 report different statistical parameters, making comparison of results partially difficult. Please, be consistent in the presentation of results.

*R: We thank the reviewer for this helpful observation. We acknowledge that Table 6 (training phase) and Table 8 (testing phase) present different sets of statistical parameters. This difference stems from the distinct analytical levels at which each evaluation was performed:*

- *Table 6 summarizes cross-validation performance at the frame level, where Specificity and NPV are not directly interpretable due to the multi-frame sampling structure;*
- *Table 8 presents external test results at the audio level, where all confusion matrix–derived metrics (including Specificity and NPV) can be consistently computed.*

*To address this comment, we have harmonized the presentation of both tables by:*

- *Clarifying in the legends that the metrics differ due to evaluation level (frame vs. audio);*
- *Adding an explanatory note in the Methods (Statistical Analysis) section detailing why certain metrics were not computed at the training stage;*
- *Ensuring consistent order and formatting of all metrics reported across both tables (Recall, Specificity, PPV, NPV, Accuracy, F1-score, AUC).*
- *These adjustments improve readability and ensure full transparency regarding metric computation and interpretability.*

______________________________________________________________________
