# Supplementary material for: ML-based detection of depressive profile through voice analysis in WhatsApp™ audio messages of Brazilian Portuguese Speakers
Source: PLOS Ment Health. 2026 Jan 21;3(1):e0000357. doi: 10.1371/journal.pmen.0000357 (PMC12822941; doi:10.1371/journal.pmen.0000357)
Supplement: Attachment — Submitted filename: RL_DepressionVoice_29october.docx [file pmen.0000357.s007.docx]

**Prof. Lucas Murrins Marques, PhD**

Mental Health Department, Santa Casa de São Paulo School of Medical Sciences, São Paulo, SP, Brazil.

Dona Veridiana Street 55, 3o. Floor, Higienópolis – São Paulo / SP, Brazil

ORCID: 0000-0003-2999-2448

Email: [lucas.murrins@fcmsantacasasp.edu.br](mailto:lucas.murrins@fcmsantacasasp.edu.br)

**ML-Based Detection of Depressive Profile Through Voice Analysis in WhatsApp™ Audio Messages of Brazilian Portuguese Speakers**

Dear Editorial team,

We sincerely thank the Academic Editor, Dr. Ariel Soares Teles, and the reviewer for the positive and supportive feedback on our revised manuscript.

We are very pleased that the reviewer confirmed that all previous points have been addressed and that the paper is now technically sound and ready for publication.

In this brief revision, we have addressed the minor remaining comment regarding the inclusion of supporting references for the statements related to masculine norms (lines 497–501).

**Sincerely,**

**Lucas Murrins Marques**

______________________________________________________________________

**Reviewers' comments:**

**Reviewer #3:**

Minor Concerns:

1) The only thing I ask is to add some reference to the claims related to the ‘masculine norms’ (lines 497–501).

*R:* We thank the reviewer for this valuable suggestion.

We have now incorporated appropriate references to support our discussion on how masculine norms influence emotional expression and help-seeking behavior in men, potentially shaping acoustic manifestations of depressive speech.

Specifically, we added the following citations:

- Addis, M. E., & Mahalik, J. R. (2003). Men, masculinity, and the contexts of help seeking. American Psychologist, 58(1), 5–14.
- Seidler, Z. E., Dawes, A. J., Rice, S. M., Oliffe, J. L., & Dhillon, H. M. (2016). The role of masculinity in men’s help-seeking for depression: A systematic review. Clinical Psychology Review, 49, 106–118.
- Rice, S. M., Kealy, D., Oliffe, J. L., & Seidler, Z. E. (2023). Men, masculinities, and mental health: The need for evidence-informed clinical practice. The Lancet Psychiatry, 10(3), 197–209.

The revised sentence in the manuscript (lines 497–501) now reads:

“*Social and cultural factors—particularly traditional masculine norms—may also influence vocal expressivity and emotional communication, contributing to flatter prosody and reduced pitch variability among men (Addis & Mahalik, 2003; Seidler et al., 2016; Rice et al., 2023). These tendencies can attenuate the acoustic cues associated with depressive affect, helping explain the lower classification accuracy observed in male participants.*”

We believe this addition strengthens the theoretical grounding of the manuscript and appropriately contextualizes the gender-related findings*.*

______________________________________________________________________
